# Immune checkpoint inhibitor-related pancreatitis: What is known and what is not

**DOI:** 10.1515/med-2023-0713

**Published:** 2023-06-02

**Authors:** Chunyan Jiang, Wen Tang, Xu Yang, Hongwei Li

**Affiliations:** Department of Internal Medicine and Geriatrics, Beijing Friendship Hospital, Capital Medical University, Beijing, China; Department of Nuclear Medicine, Beijing Friendship Hospital, Capital Medical University, Beijing, China; Department of Cardiology, Cardiovascular Center, Beijing Friendship Hospital, Capital Medical University, No. 95 Yong’an Road, Xicheng District, Beijing 100050, China; Beijing Key Laboratory of Metabolic Disorder Related Cardiovascular Disease, Beijing, China

**Keywords:** immune checkpoint inhibitor, PD-I inhibitor, sintilimab, pancreatitis, adverse event

## Abstract

A 47-year-old man presented with a 1 week history of progressive fatigue and decreased appetite. He had stage IV oral squamous cell carcinoma and was treated with sintilimab, a programmed cell death protein 1 inhibitor, 22 months earlier. Laboratory work-up revealed significant elevation of bilirubin, liver enzymes, glucose, and lipase. Ultrasound examination and magnetic resonance cholangiopancreatography showed severe stenosis and occlusion of the pancreatic segment of the common bile duct, and PET/CT revealed swelling of the pancreas with diffuse increase in glucose metabolism. He was diagnosed with immune checkpoint inhibitor (ICI)-related pancreatitis and the treatment with sintilimab was permanently discontinued. He was administered systemic methylprednisolone at a dose of 2 mg/kg/day and subcutaneous insulin injection, without intravenous fluid or protease inhibitor. He improved quickly and received oral methylprednisolone for 10 months in gradually decreasing doses. He maintained well at 20 month follow-up. ICI-related pancreatitis is rare and varied. Further studies are needed to investigate the differences in the two types of ICI-related pancreatitis: acute pancreatitis and autoimmune pancreatitis-like cases.

## 1 Introduction

Immune checkpoint inhibitors (ICIs) against cytotoxic T lymphocyte-associated antigen 4 and programmed cell death protein 1 (PD-1) and its ligand PD-L1 have achieved prominent efficacy in the treatment of many types of cancers. However, ICIs are associated with a series of immune-related adverse events [1,2], which should be alerted by both physicians and patients. Compared with ICI-related dermatological, gastrointestinal, pulmonary, or endocrine toxicities, ICI-related pancreatitis is rare and varied, and no routine monitoring or optimal treatment has yet been established. Here we report a case of autoimmune pancreatitis-like ICI-related pancreatitis that presented with obstructive jaundice, elevated pancreatic enzyme (lipase), and hyperglycemia, but without abdominal pain and was successfully treated with systemic methylprednisolone alone.

## 2 Case report

A 47-year-old man presented with a 1 week history of progressive fatigue and decreased appetite. He reported no fever, vomiting, or abdominal pain. He had a 40 month history of stage IV oral squamous cell carcinoma and had been treated with sintilimab, a PD-1 inhibitor, at 200 mg every 3 weeks for 22 months. He experienced mild rash and pruritis, which could be alleviated by topical corticosteroids. Blood and urine tests were monitored intermittently and had been maintained normal within the initial 8 months. However, the monitoring was discontinued 14 months ago for personal reason. He received the last dose of sintilimab 8 days before the admission, and denied any other medical history or medications.

**Figure 1:**
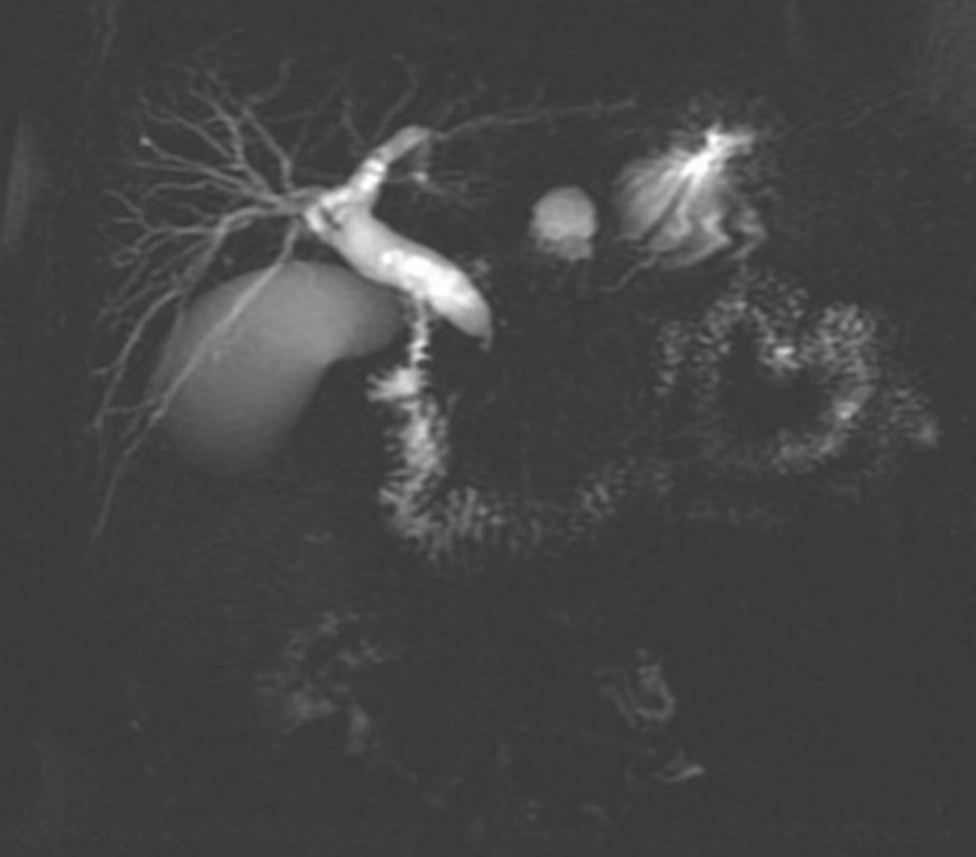
MRCP shows severe stenosis and occlusion of the pancreatic segment of the common bile duct and dilation of the intrahepatic and extrahepatic bile ducts.

**Figure 2:**
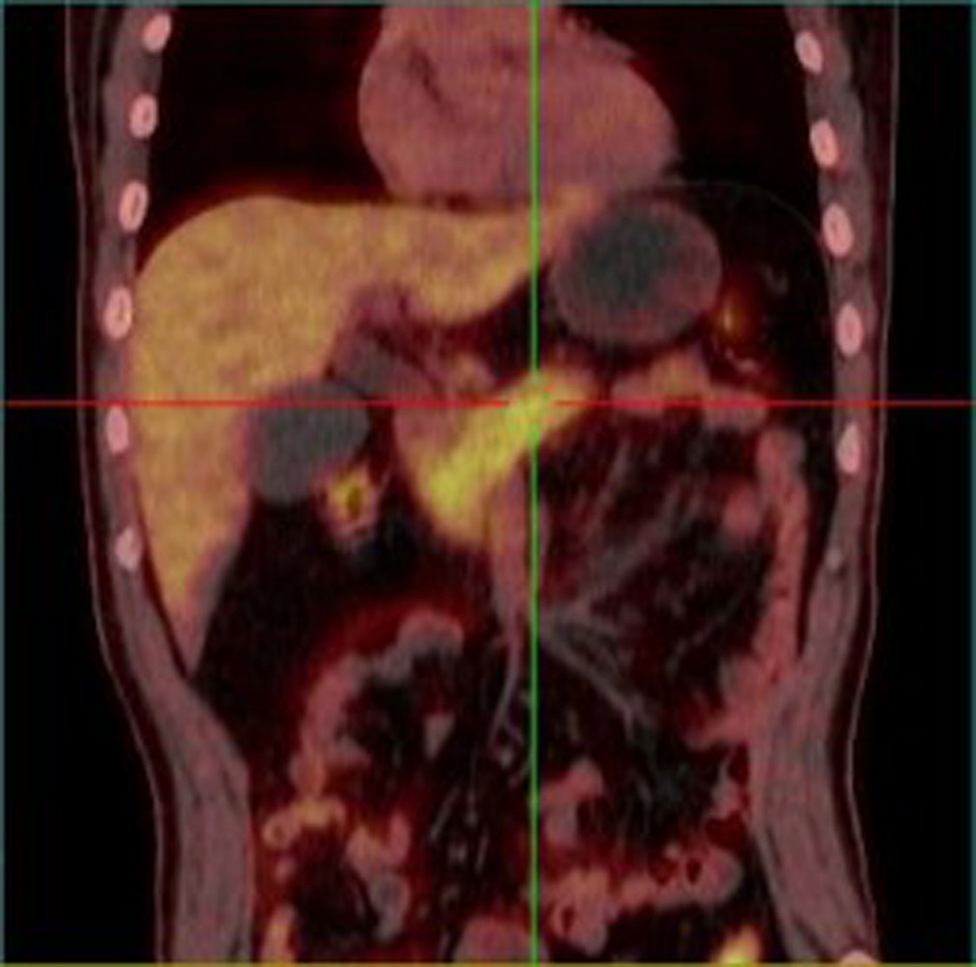
PET/CT reveals swelling of the pancreas with diffuse increase in glucose metabolism, especially in the head and neck region.

Physical examination was notable for jaundice, without abdominal tenderness or hepatosplenomegaly. Laboratory work-up revealed normal white blood cell counts (6,290/μL; reference range 3,500–9,500) and C-reactive protein level (1 mg/L; reference range <8) but significant elevation of total bilirubin (127.08 μmol/L; reference range 3.42–21.00), direct bilirubin (79.25 μmol/L; reference range <8.60), indirect bilirubin (47.83 μmol/L; reference range <12.00), alanine aminotransferase (812 U/L; reference range 9–50), aspartate aminotransferase (354 U/L; reference range 15–40), alkaline phosphatase (667 U/L; reference range 45–125), gamma-glutamyltranspeptidase (2,315 U/L; reference range 8–55), glucose (18.21 mmol/L; reference range 3.92–6.16), HbA1c (6.96%; reference range 4.27–6.07), and lipase (272 U/L; reference range 13–60). Urine test was positive for protein and red blood cells (687.7/μL; reference range <25), with 80% abnormal erythrocytes. The levels of immunoglobulin G (IgG), IgM, IgA, and IgG subtypes were normal, and detection of autoantibodies was negative. Lymphocyte subsets showed low T lymphocyte (CD3+) ratio (52.28%; reference range 58.6–83.1), high B lymphocyte (CD19+) ratio (25.96%; reference range 3.5–15.4), and low CD4/CD8 ratio (0.47; reference range 0.7–2). Ultrasound examination and magnetic resonance cholangiopancreatography (MRCP) showed severe stenosis and occlusion of the pancreatic segment of the common bile duct and dilation of the intrahepatic and extrahepatic bile ducts (Figure 1). Position emission tomography/computed tomography (PET/CT) revealed no evidence of recurrence or metastasis but slightly swelling of the pancreas with diffuse increase in glucose metabolism, especially in the head and neck region (Figure 2). Further investigations revealed normal response of C-peptide in oral glucose tolerance test and negative anti-islet cell and anti-glutamic acid decarboxylase antibodies.

The patient refused biopsy. The treatment with sintilimab was permanently discontinued and the patient was administered intravenous methylprednisolone at a dose of 2 mg/kg/day and subcutaneous insulin injection, without fasting or protease inhibitor. On the next day of the treatment, laboratory investigations showed significant improvement. Over the course of 2 weeks, liver function turned to be normal (Table 1). The patient continued the treatment of oral methylprednisolone at 40 mg/day and subcutaneous insulin at discharge. No other anti-diabetes treatment was performed. At outpatient follow-up 2 weeks later, ultrasound examination revealed normalized bile duct imaging. He received oral methylprednisolone for a total treatment period of 10 months in gradually decreasing doses and maintained well at 20 month follow-up.

**Table 1:** Laboratory tests before and after treatment with methylprednisolone and insulin

Time	ALT (U/L)	AST (U/L)	TBIL (μmol/L)	DBIL (μmol/L)	IBIL (μmol/L)	ALP (U/L)	GGT (U/L)	Glucose (mmol/L)	Amylase (U/L)	Lipase (U/L)	CD4/CD8
2021-8-2^a^	812	354	127.08	79.25	47.83	667	2,315	18.21	44	272	0.49
2021-8-6^b^	745	338	126.04	87.05	38.99	643	2,285	12.94	51	303.9	—
2021-8-9^c^	553	193	65.05	29.98	35.07	570	2,056	10.05	13	44.3	—
2021-8-11	509	171	42.68	20.30	22.38	354	1,296	4.57	9	17.4	0.73
2021-8-13	463	98	38.05	17.12	20.93	311	1,092	5.02	26	32.1	—
2021-8-16	212	30	27.23	13.07	14.16	212	732	5.14	28	28.9	0.68
2021-8-26	52	19	24.07	9.37	14.70	114	404	4.72	26	30.0	—
2021-9-2	37	22	23.93	7.06	16.87	105	248	4.88	22	25.6	0.80
2021-9-9	30	21	20.02	5.01	15.01	79	159	5.31	23	14.4	—
2021-9-30	34	20	12.34	2.66	9.68	73	55	5.53	24	27.4	—
2021-11-1	23	21	10.66	2.31	8.35	67	47	4.60	20	23.6	—
2021-12-8	26	24	12.98	2.83	10.15	62	48	5.20	27	36.4	—
2022-3-28	20	24	13.38	2.38	11.00	70	21	5.01	20	30.6	—
2022-7-1^d^	20	25	10.80	2.15	8.65	60	22	5.54	16	33.0	—
2023-1-4	30	29	12.12	4.14	7.98	89	29	6.05	18	14.4	
Reference range	9–50	15–40	3.42–21	0–8.6	0–12	45–125	8–55	3.92–6.16	35–135	13–60	0.7–2

Abbreviations: ALT, alanine aminotransferase; AST, aspartate aminotransferase; TBIL, total bilirubin; DBIL, direct bilirubin; IBIL, indirect bilirubin; ALP, alkaline phosphatase; GGT, glutamyl transpeptidase; CD, cluster of differentiation. ^a^The day at admission. ^b^The 4th day after sintilimab discontinuation and insulin treatment. ^c^The next day after methylprednisolone treatment at a dose of 2 mg/kg/day. ^d^One month after methylprednisolone discontinuation.


**Informed consent:** Obtained from the patient.

## 3 Discussion

ICI-related pancreatic adverse events include elevated amylase/lipase, pancreatitis, hyperglycemia, and exocrine pancreatic insufficiency [3]. According to a recent systematic review, the incidence of asymptomatic lipase elevation after ICI use is 2.7% and grade 2 pancreatitis is 1.9% [4]. According to the Common Terminology Criteria for Adverse Events version 5.0, the pancreatitis in the present case is classified as grade 3. Current guidelines for ICI-related adverse events either do not mention lipase/amylase elevation [1,5], or do not recommend routine lipase/amylase monitoring in asymptomatic patients unless pancreatitis is clinically suspected [2,6], or do recommend regular lipase monitoring during treatment with ICIs [7].

Making an accurate diagnosis of ICI-related pancreatitis is clinically challenging because the clinical manifestations may be subtle. In the present case, the diagnosis of ICI-related pancreatitis and diabetes-induced by pancreatitis could be confirmed based on the laboratory analyses and imaging studies, even without symptoms of abdominal pain. After the application of drugs based on immunomodulation, antibodies are created that can damage various organs such as the kidney and lead to a fatal outcome [8]. However, no abnormal antibodies were detected in serum in this case.

According to limited case reports regarding pancreatitis due to ICIs, there are two phenotypes of ICI-related pancreatitis: acute pancreatitis and autoimmune pancreatitis (AIP)-like cases [9]. The differences in these two types might aid in our understanding the mechanisms underlying the development of ICI-related pancreatitis. With the absence of characteristic symptoms of epigastric pain, normal serum C-reactive protein and IgG4, as well as the imaging characteristics, the present patient is more likely to be consistent with type 2 AIP [10]. According to the four types of ICI-related diabetes (acute autoimmune insulin-dependent diabetes, type 2 diabetes-like phenotype, diabetes induced by AIP, and diabetes following autoimmune lipoatrophy), the new-onset hyperglycemia with an almost normal response of C-peptide in oral glucose tolerance test and negative anti-islet cell and anti-glutamic acid decarboxylase antibodies was diagnosed as diabetes induced by AIP, but not type 1 or type 2 diabetes [3].

No optimal treatment for ICI-related pancreatitis has yet been established. The National Comprehensive Cancer Network Guideline recommends that for grade 3–4 pancreatitis (severe and life-threatening), immunotherapy should be permanently discontinued, and treatment with 1–2 mg/kg/day glucocorticosteroids and intravenous fluids should be started [7], whereas other guidelines offer no recommendations [1,2]. The differences in management and prognosis of the two types of ICI-related pancreatitis are not clear. The present patient received systemic glucocorticosteroid alone, without intravenous fluids or proteinase. Although most ICI-related pancreatitis are clinically well manageable by use of systemic glucocorticoids, as in the present case, fatal ICI-related pancreatitis has been reported [11]. Timely patient and physician education, careful evaluations including systemic physical examination and appropriate laboratory tests before each dose of immunotherapy, familiarity with ICI-related pancreatic injury characteristics by the clinicians, appropriate management protocol of immune-related pancreatic injury, self-surveillance, and outpatient follow-up are vital factors affecting patient outcomes. Further studies are needed to investigate the differences in mechanism, pathology, management, and prognosis of the two types of ICI-related pancreatitis.
